# Anabolic-Androgenic Steroid Misuse: Mechanisms, Patterns of Misuse, User Typology, and Adverse Effects

**DOI:** 10.1155/2021/7497346

**Published:** 2021-12-10

**Authors:** Jack B. Ding, Marcus Z. Ng, Steven S. Huang, Mark Ding, Kevin Hu

**Affiliations:** ^1^University of Adelaide, Adelaide 5005, Australia; ^2^Division of Medicine, Lyell Mcewin Hospital, Adelaide 5112, Australia

## Abstract

Anabolic-androgenic steroids (AAS) encompass a broad group of natural and synthetic androgens. AAS misuse is highly prevalent on a global scale, with the lifetime prevalence of AAS misuse in males being estimated to be around 6%, with 15 to 25% of male gym attendees using it at any one time. AAS are associated with sudden cardiac death, neuropsychiatric manifestations, and infertility. The average AAS user is unlikely to voluntarily declare their usage to a physician, with around 1 in 10 actively engaging in unsafe injection techniques. The aim of this paper is to review the current evidence base on AAS with emphasis on mechanisms of action, adverse effects, and user profiles that are most likely to engage in AAS misuse. This paper also reviews terminologies and uses methods specific to the AAS user community.

## 1. Background and Introduction

Anabolic-androgenic steroids (AAS) are more commonly referred to as anabolic steroids and encompass a diverse group of both synthetic and endogenous androgens [1]. Testosterone, an endogenous androgen, was isolated and characterized in 1935 and subsequently made available for synthesis and exogenous administration [2]. Since then, substantial research and development have led to the creation of numerous testosterone derivatives with structural alterations that result in properties unique to each derivative. The earliest use of AAS was in the context of competitive sport, including its high profile utilization by the 1954 Russian weight lifting team at the Olympics [3]. With the subsequent creation of international antidoping agencies such as the World Anti-Doping Agency and explicit banning of AAS use by the International Olympics Committee in 1967, its use among elite athletes has attenuated. Instead, the use of AAS has become more pervasive among users at a nonelite level, with a 2014 meta-analysis of 187 studies estimating the lifetime prevalence of AAS use in men to be around 6% and a 2005 paper suggesting its prevalence amongst male gym attendees to be around 25% [4, 5]. However, the average clinician may not encounter a significant number of clinical presentations related to AAS use for several reasons. First, the most devastating adverse effects of AAS such as an increased risk of premature death from all causes or twice-fold increased cardiovascular mortality rate manifest suddenly and definitively [6]. Second, AAS consumers are rarely proactive with disclosing their use status with their physicians, with one study finding that 56% of AAS users had never informed any physician that they use or have used AAS [7]. Alarmingly, this is despite 1 in 10 reporting unsafe injection techniques and close to 100% reporting subjective side effects [5]. The primary reason behind this underreporting has been suggested by some to be due to a universal sense of distrust among AAS users regarding the knowledge the average physician has on AAS [8]. Further, physicians rarely specifically inquire about AAS use during history taking, thereby missing an opportunity to establish rapport about the topic [7]. Indeed, anecdotal reports of advice given by clinicians inaccurately purporting that AAS are ineffective for muscle growth have surfaced, with some commentors arguing that such blanket statements can prematurely sever any chance of developing rapport and generating critical discussion with AAS users [8]. Instead, commentors have suggested that an in-depth understanding of AAS science and mechanisms is necessary for the establishment of foundational discussion with AAS users, which can provide a physician with a footing to later effectively pivot to conversations regarding AAS discontinuation [8]. While there is an abundance of literature pertaining to the side effects of AAS use, there is a paucity of reviews that discuss the motives, efficacy, and patterns of AAS misuse. This review aims to fill this gap while also refreshing and updating the physician about the underlying basic science of AAS and the prognostic implications of AAS use.

## 2. A Review of Testosterone

The primary function of the testes is to produce testosterone and maintain an environment optimized for spermatogenesis [9]. These dual functions are coordinated by the overarching hypothalamic pituitary gonadal (HPG) axis. The first step of the axis involves the pulsatile release of gonadotropin releasing hormone (GnRH) from the hypothalamus, which reaches the anterior pituitary through the hypophyseal-portal system [10]. The anterior pituitary is stimulated by GnRH and in result secretes follicle-stimulating hormone (FSH) and luteinizing hormone (LH) into the systemic circulation. FSH acts on Sertoli cells in the seminiferous tubules, where it promotes spermatogenesis. LH targets Leydig cells located adjacent to the seminiferous tubules, where testosterone production is stimulated [11]. Notably, the intratesticular testosterone concentration is typically 50–100 times higher than the serum concentration of testosterone [12]. Intratesticular testosterone helps optimize spermatogenesis, and the steep concentration gradient helps maintain systemic testosterone levels, where it exerts additional effects on androgen sensitive tissues.

The classic effects of serum testosterone derive from its interaction with the androgen receptor. Testosterone binding to the intracytosolic receptor leads to its transport to the nucleus, where it directly regulates gene transcription and protein synthesis [13]. An androgen is defined as any natural or synthetic steroid that binds to the androgen receptor that results in the stimulation of postreceptor functions [14]. The action of testosterone does not chiefly revolve around the androgen receptor modulation. Under physiologic circumstances, there is a constant stream of prereceptor, receptor, and postreceptor mechanisms [13]. The primary prereceptor modulations of serum testosterone involve a portion of it being converted into 17 *β*-estradiol by aromatase and another portion into dihydrotestosterone (DHT) by 5*α*-reductase [15]. Since adipose tissue contains a significant amount of aromatase, obesity can result in increased levels of 17 *β*-estradiol due to increased conversion of testosterone by aromatase [16]. Nandrolone (19-nortestosterone) is an endogenously produced steroid that exists as an intermediate in the process by which aromatase converts testosterone to estradiol. 19-Nortestosterone derivatives are one of the main classes of AAS. Under physiologic circumstances, 19-nortestosterone levels are not present in the bloodstream in appreciable quantities [17]. Nandrolone differs from other AAS in that it is also a potent progestogen that exhibits relatively lesser androgenic effects in tissues such as the prostate and scalp [18]. It is an agonist of both the androgen receptor and the progesterone receptor. Its progestogenic properties result in substantial antigonadotropic activity, resulting in additional suppression of the HPT axis [19]. DHT is a more potent androgen than testosterone, exhibiting over 3x higher affinity for the androgen receptor. The physiologic functions of DHT differ from testosterone in that it primarily signals through intracine and paracrine mechanisms in tissues where 5-alpha reductase is concentrated, exhibiting targeted local effects in contrast to the broader overarching effects of testosterone [20]. An example of this is the prostate, where its levels are up to 10-fold higher than serum testosterone, due to high local concentrations of 5-alpha reductase [21]. Unlike testosterone, DHT derivatives such as androstanolone cannot be targeted by aromatase, and therefore administration of high doses of exogenous DHT poses minimal risk for estrogenic side effects [22]. Despite DHT being described as a potent androgen receptor agonist, it has very weak anabolic effects. This is likely because 3a-hydroxysteroid dehydrogenase, an enzyme concentrated in skeletal muscle, has high affinity for DHT derivatives and converts it into metabolites that have very weak affinity for the androgen receptor [23]. Due to all this, the role DHT plays as a circulating androgen is likely minor compared to testosterone. Indeed, the serum concentrations of DHT do not necessarily correlate with its intracellular concentrations [15].

Under normal physiologic circumstances, androgen receptors are saturated by endogenous testosterone, even if the levels are at the lower range of normal for males. This detail contributed to decades of debate as to whether supraphysiologic doses of testosterone stimulate additional anabolic effects on muscle size and strength [24]. The current consensus is that androgenic anabolic steroids do indeed improve both. One study randomized 61 eugonadal men into five groups, with each receiving various doses of exogenous testosterone for 20 weeks. By the end of the trial, the men who received 600 mg injections gained on average 7.9 kg of lean muscle mass and lost about 1 kg of body fat. Remarkably, participants were specifically instructed not to engage in any strength training or moderate or heavy aerobic exercise routines during the period of study, with this request being reiterated every 4 weeks [25]. Since androgen receptors are normally saturated by physiologic levels of testosterone, yet supraphysiologic doses of testosterone can increase muscle mass and body strength, the anabolic effects of exogenous testosterone may be explained by mechanisms other than androgen receptor modulation [26]. One possibility involves modulation of the glucocorticoid receptor. Animal studies have shown that exogenous testosterone is effective at preventing atrophy secondary to glucocorticoid use [27]. Human studies have shown that AAS can displace glucocorticoids bound to glucocorticoid receptors [28]. Since glucocorticoids exert profound antagonistic effects on skeletal muscle anabolism, suppression of its action at the receptor level may result in an overall increase in muscle mass [29]. Inhibition of glucocorticoid activity likely results in activation of the growth-hormone (GH) and insulin-like growth factor (IGF-1) axis, where it usually has a suppressive effect. Both IGF-1 and GH are considered ‘anabolic giants' in the context of skeletal muscle growth and repair, and thus an intensification of their effects could potentially explain some of the massive skeletal muscle gains seen with AAS use [30]. Other studies have shown that AAS administration can result in androgen receptor downregulation. Since androgens can also competitively bind to estrogen receptors, if the androgen receptors are downregulated, they can interact with estrogen receptors and through that exert an antiestrogenic effect [26]. To clarify, this is not to say that AAS administration lowers estrogen levels. Rather, one study found that, over 26 weeks of exogenous anabolic steroid administration, their male participants demonstrated a 2.3-fold increase in serum testosterone concentration. Conversely, the estradiol levels of the subjects rose by around 7-fold, to approximately the normal range for females, with a reciprocal decrease in FSH and LH levels. 80% of their participants developed gynecomastia, likely as a result of the estradiol increase [31]. The significantly elevated estradiol levels can be explained by the normal activity of aromatase, which converts androstenedione to estrone and testosterone to estradiol [32]. At supraphysiologic testosterone levels, a greater amount of estradiol would be produced through this mechanism.

The satellite effects testosterone and other AAS have on other hormones such as estradiol or progesterone differ depending on the type of AAS. The variation is due to differences in chemical properties and ultimately leads to different adverse effect profiles between each type. The structural modifications synthetic AAS have on endogenous testosterone were likely designed with specific intentions in mind, such as maximizing anabolic effects while simultaneously minimizing androgenic properties [33]. Examples of structural modifications to the basic testosterone structure include the addition of a benzyl, hydroxyl, methyl, or ethyl at one or more sites [34]. ‘Designer steroids' refers specifically to anabolic steroids that are considered to have been designed and synthesized with the explicit intention of evading doping tests in competitive sport [35]. While records of designer steroids are elusive by nature, one example of their existence is epitestosterone propionate, which was manufactured as part of the former German Democratic Republic's (GDR) state funded doping program [36].

### 2.1. Typology of the AAS User

As with any illicit substance user base, the AAS community is very diverse in terms of motivations and use patterns and can be difficult to categorize. Still, attempts have been made to identify typical typologies of users, as the findings may be important for shaping public health policy. Notably, qualitative stratification is not specific to AAS; studies have been applied to the opioid and alcohol misuse populations for instance [37, 38]. Christiansen and colleagues proposed a classification method in 2016, based on a review of international literature and each authors' research experience with AAS misusers [39]. They proposed four classifications: (1) the Expert type; (2) the YOLO type; (3) the Athlete type; and (4) the Wellbeing type. In their synthesis of these typologies, they focused on a two-dimensional perspective of AAS use, which is effectiveness and perceived risk.

Christiansen and colleagues described “the Expert type” as taking a scholarly approach to AAS, their use patterns are measured, and the risks they take are generally measured. The “YOLO type” (You Only Live Once) is in some ways the archetypal opposite in that their actions are frequently spontaneous and are often motivated by rapid progress. The “Athlete type” is primarily engaged in competitive sports and is motivated by succeeding in their niche, and finally the “Wellbeing type” centralizes on physical aesthetics and tends to exhibit lower risk use strategies [39].

Zahnow and colleagues furthered these proposed typologies in 2018 by using cluster analysis followed by multinomial logistic regression on survey data from a sample of 611 AAS users across Wales, England, and Scotland [40]. They discovered four distinct categories within their data, each seeming to correspond to four typologies proposed by Christiansen. Specifically, 11% of respondents seemed to correspond to the “YOLO type,” 38% to the “Wellbeing type,” 25% to the “Athlete type,” and 25% to the “Expert type.” To our knowledge, this study is the first and latest attempt at quantifying a typographic analysis of AAS users. Importantly, it has substantiated the notion of distinct subclasses within the AAS user base and that AAS users are diverse in motives and use patterns. Overall, however, our current typographic understanding of AAS users is in a relative infancy, especially since that the population analyzed is limited to the United Kingdom and that other typologies may exist and are yet to be discovered and conceptualized.

### 2.2. Classes of AAS Compounds

Broadly, AAS compounds may be classified into three main groups, testosterone esters, 19-nortestosterone derivatives, and dihydrotestosterone derivatives, and may be further stratified based on route of consumption (oral versus intramuscular) [41, 42].  Table 1 provides an overview of these three classes and some of their common trade names.

### 2.3. Basic AAS Use Methods and Nomenclature

Most AAS regimens include one or more compounds from Table 1 taken at a supraphysiologic dose. Physiologic levels of serum testosterone in adult males range from 300 to 1,000 ng/dL, with up to 11.0 mg testosterone produced per day, whereas normal serum DHT ranges from 50 to 74 ng/dL [15, 43]. For perspective, one study found that over half of AAS users were administered weekly doses of androgens exceeding 1000 mg [5].

A review by Graham et al. identified several key nonscientific AAS use methods and terms to describe them among various sources in the bodybuilding community [44]. These terms and their interpretation are tabulated in Table 2.

## 3. Performance-Enhancing Drugs (PEDs) in the Context of AAS Use

AAS regimens frequently include one or more ancillary drugs to augment the efficacy of the primary AAS or to offset any undesirable side effects [45]. Performance-enhancing drugs (PEDs) in the context of AAS use characterize multiple classes of supplementary compounds that have distinct hormonal interactions.  Table 3 provides an overview of popular PEDs and their intended effects and side effects.

### 3.1. Performance-Enhancing Drugs in the Postcycle Period

The term ‘postcycle' describes the time immediately following an AAS cycle when users typically encounter a myriad of side effects, such as gynecomastia and hypogonadism secondary to low endogenous testosterone levels and or loss of exogenous testosterone supply following cycle completion [49]. Counteracting AAS side effects is one popular use case of PEDs in the bodybuilding community, with a 2018 survey of 231 AAS users finding that 56% of participants reported that they had engaged in some form of postcycle therapy [50].

Karavolos et al. used a systematic online search process to investigate the various websites and forum communities where participants were able to request and or deliver advice pertaining to counteracting AAS induced side effects. They found that up to a third of the identified websites claimed to sell AAS and or products intended to combat AAS side effects without a prescription. Personal anecdotes and recommendations from anonymous participants formed the bulk of advice on the discussion forums. Scientific publications were cited by participants, though they were frequently taken out of context or were ungeneralizable to the AAS user population [46].

Table 4 represents commonly encountered side effects in the “postcycle” period and strategies frequently advocated to offset them in the AAS user community.

### 3.2. Adverse Effects of AAS Use

The adverse effect profile of exogenous AAS administration is a source of contention, with some raising the possibility that interpretations of data had been exaggerated [56]. A 2010 review concluded that the prevalence of somatic and psychiatric adverse effects was limited and that the typical user was given AAS with the intention to augment their sporting accomplishments [57]. Conversely, others have suggested that AAS misuse is a public health issue and that the widespread presence of androgen receptors in the body is one possible explanation for AAS side effects being pervasive across multiple organs and systems [26].

Among the complications of AAS use, cardiovascular side effects have been postulated to have the strongest mortality altering consequences, with some studies suggesting it to be the root cause of premature death in AAS users in up to 33–66% of cases, with the remaining being mainly caused by liver failure, cancer, and suicide [58]. A 2019 retrospective study of 545 AAS users reported mortality rates to be around three times higher compared to matched male controls (95% CI 1.3–7.0) [59]. In addition, the AAS cohort was found to be significantly more likely to present to be admitted to hospital compared to the control group. In a postmortem review, an analysis of 19 cases in which autopsy had excluded extracardiac mechanisms of death revealed left ventricular hypertrophy with concomitant fibrosis to be the most common finding [60]. These pathologic findings corroborate with clinical findings, such as significantly reduced left ventricular systolic and diastolic function, as reported in a 2017 cross-sectional study of 140 weightlifters [61]. While the mechanisms behind cardiac death in AAS users are incompletely understood, four possibilities have been proposed, which include (1) atherosclerosis potentiation model, (2) thrombosis model, (3) coronary vasospasm, and (4) direct cardiac injury [62]. The stimulatory effect of exogenous AAS administration on hematopoiesis theoretically disposes individuals to increased risk of thrombosis, though the current evidence body for this is limited to case repots and epidemiological studies [63].

Psychiatric measures as scored in the Hostility and Direction of Hostility Questionnaire (HDHQ) and Symptoms Check List-90 (SCL-90) have been associated with AAS use, with symptom intensity correlating with the severity of misuse [64]. Hypomanic or manic syndromes have also been described as two possible manifestations, with irritable ‘roid rage' being the classic lay term that describes aggressive behavior that is seemingly due to AAS use [65]. Pope et al. assessed psychiatric outcome measures between a placebo group and a group that received exogenous testosterone. They concluded that the AAS group had significantly higher scores on the Young Mania Rating Scale (YMRS) (*p*=0.002), with 16% of the group being classified as mildly or markedly hypomanic [66]. AAS may also dispose users to impaired cognitive functions or structural brain alterations, with one study that conducted structural MRI brain imaging on AAS users and nonusers noting a negative association between cortical thickness and brain volume and AAS use [67]. Notably, this is in the context of an association between AAS use and poorer performance on cognitive tests [68, 69]. Furthermore, some commentators have raised the possibility of a link between supraphysiologic testosterone levels and dementia through androgenic induction of additional oxidative stress [70].

Infertility following AAS use is secondary to suppression of intratesticular testosterone levels, which can lead to azoospermia or oligozoospermia [71]. It is generally thought that restoration of sperm count and quality after AAS cessation would eventuate with sufficient passage of time, with the 12- to 24-month mark being cited as the timeframe for the bulk of AAS users [72, 73]. One 2020 cross-sectional study of 72 AAS misusers and 31 healthy controls noted no significant difference in sperm output, sperm concentration, and sperm motility between former AAS users and nonusers, with the mean recovery time for these parameters post-AAS cessation being 14 months, 10 months, and 37 months, respectively [74]. Conversely, a 2021 prospective study of AAS users who intended to initiate a cycle discovered that at 1 year follow-up, 34% of users had a total sperm count below 40 million (normal range ≥39 million) [75]. However, this study followed up participants based on when they were starting AAS use, rather than stopping it, which would provide a more generalizable conclusion. An overarching limitation of the interpretation of all of these studies is that restoration of fertility is defined by the restoration of reproductive physiologic parameters, rather than real-world world outcomes, such as the time to achieve pregnancy. A 2021 Danish study of 545 males who returned an AAS positive result on a blood test discovered that, in the decade prior to the positive result, the AAS group had a significant lower fertility rate compared to the healthy control group, with a rate ratio of 0.74 (95% CI: 0.60–0.90, *p*=0.0028). In the years after AAS cessation, the AAS group still exhibited a 7% lower total fertility rate compared to control, though there was no significant group difference for rates of assisted reproduction [76]. Therefore, the current literature tentatively suggests that reproductive recovery may be possible for most former AAS users. However, further research investigating real-world world outcomes, such as the time to achieve pregnancy, is required to deliver more substantiative conclusions.

Hepatotoxicity is a commonly encountered side effect of AAS use, with one study noting AAS related liver injury comprised 8% of their total cases of drug-induced liver injury [77]. Notably, only 17*α*-alkylated AAS appears to have hepatotoxic properties. While the underlying mechanism behind this is yet to be fully elucidated, increased oxidative stress secondary to increased mitochondrial *β*-oxidation has been hypothesized to play a role [78]. The spectrum of AAS induced liver injuries is diverse in possible pathology. Schwingel and colleagues analyzed 182 asymptomatic Brazilian AAS users and reported that 38% had raised liver markers, 12% had hepatic steatosis, and there was one case each of hepatocellular adenoma, focal nodular hyperplasia, hepatitis B, and hepatitis C [79]. While hepatocellular adenomas are typically benign solitary masses, cases have been described where multiple adenomas had formed in the setting of AAS use and resulted in the patients suffering from hemorrhagic shock that nearly resulted in death [80, 81]. There are also case reports of hepatocellular carcinoma (HCC) forming in the setting of AAS use, noting that approximately 4% of hepatocellular adenomas transform to HCC [82]. The mechanisms and association between AAS and hepatic cell proliferation are worthwhile of further exploration.

AAS related renal damage is less studied compared to other adverse effects, although some possible mechanisms include direct toxicity or bile acid nephropathy secondary to AAS related liver injury [26, 83]. The spectrum of possible AAS related kidney injuries is diverse, ranging from transient fluctuations in creatinine levels to end-stage chronic kidney disease or secondary focal-segmental glomerulosclerosis *d* [84].

## 4. Conclusions

Anabolic-androgenic steroids include a highly varied group of synthetic and endogenous androgens, each with a unique chemical structure and adverse effect profile. Typological depictions of the anabolic-androgenic steroid base have been attempted, with some cross-sectionally derived evidence substantiating the idea that there are distinct user profiles within the user community. However, further work is needed to conceptualize other possible typologies, especially from datasets outside the United Kingdom, and this area is at a very early level of development. Further qualitative stratification may prove beneficial from a public health perspective, especially if policies and interactions at national, regional, and clinician-patient levels are able to be tailored to specific user subtype. General consumption strategies observed in AAS community include administering an exogenous testosterone for distinct periods and then tapering the drug down over a predetermined amount of time. Others combine multiple AAS or augmentative agents as part of a same regimen. Some users never cease exogenous testosterone, instead alternating between high and low doses of AAS. The level of risk stratification or spontaneity regarding consumption strategies is thought to vary between typographic subtypes.

AAS is associated with myriad adverse effects that are pervasive across multiple organ systems. Some AAS users attempt to attenuate side effects such as gynecomastia, androgenic steroid induced hypogonadism, or sexual dysfunction through self-initiating interventions such as tamoxifen, anastrozole, or hCG injections. The adverse effect profile of AAS use has historically been a source of contention, with commentators mixed on the severity of the situation. Our review shows that the adverse effects of AAS are indeed disseminated across multiple organ systems, with the true extent of it yet to be revealed by further, ideally prospective-design studies. The increased risk of premature death, development and subsequent hemorrhagic rupture of hepatic adenomas, and the possibility of remaining infertile for up to 2 years after AAS cessation are possible points for the clinician to use to dissuade individuals from misusing AAS.

## Figures and Tables

**Table 1:** An overview of selected injectable and oral anabolic steroids.

Injectable AAS		
Dihydrotestosterone derivatives	Testosterone derivatives	19-nortestosterone derivatives
Drostanolone (Masteron)	Testosterone cypionate (Test C, Testex Leo)	Nandrolone compounds (Deca Durabolin, NPPP, Pondus, Testobolin)
Mesterolone (Proviron)	Testosterone enanthate (Test E, Testoviron)	Trenbolone compounds (“Tren”)
Stanozolol (Stromba, Winstrol)	Testosterone propionate (Test Prop)	
	Testosterone decanoate	
	Boldenone undecylenate (Equipoise)	
	Sustanon 250	

Oral anabolic steroids		
Dihydrotestosterone derivatives	Testosterone derivatives	Prohormones
Oxandrolone (Anavar)	Methyltestosterone	Methasterone (superdrol)
Oxymetholone (Anadrol)	Metandienone (“Dianabol”)	1-Androstenedione (andro)
Metenolone (Primobolan)	Fluoxymesterone (Halotestin)	Dehydroepiandrosterone (DHEA)
Stanozolol (Winstrol)	Chlorodehydromethyltestosterone (Turinabol)	

**Table 2:** Common terminologies used by the AAS user community.

Terminology	Interpretation
Stacking/blending/shotgunning	Combining more than one AAS or non-AAS drug into a single regimen to be taken concurrently. This could involve mixing oral and injectable types or taking compounds intended for veterinary use.
Tapering	Gradually weaning an AAS dose down.
Plateauing	When a drug becomes ineffective at its current dose. Suggestive of the need to increase calorie intake, increase the drug dose, or stop the drug.
Cycle	Using one or more AAS for a fixed period, ranging anywhere from 6 to 16 weeks, and then stopping for approximately a similar duration of time.
Pyramiding	Gradually maximizing the dosage of an AAS and then gradually minimizing the dosage of the same drug to zero over a predefined amount of time.
Blast and cruise/bridging [45]	Alternating between periods of high and low doses of AAS, but never completely ceasing drug use. Periods of high AAS dose are a ‘blast' phase, whereas periods of lower AAS dose are a ‘cruise' phase.

**Table 3:** Selected performance-enhancing drugs used in relation to AAS.

Class	Selected formulations	Intended effects	Adverse effects
Aromatase inhibitors [46]	Anastrozole, letrozole, exemestane	Increase pituitary gonadotrophin release and therefore increasing endogenous testosterone release by reducing estrogenic negative feedback	Decreased bone density, sexual dysfunction, central adiposity
SERMs [46]	Clomiphene, tamoxifen	Increase pituitary gonadotrophin release and therefore increasing endogenous testosterone release by reducing estrogenic negative feedback	Vasomotor symptoms, visual disturbances, headaches
Fat-burning compounds	Dinitrophenol, liothyronine (T3), clenbuterol	Achieve lower body fat percentages	Hypertension, arrhythmias
Insulin	Lispro, glargine	Increase lean muscle mass	Hypoglycaemia
Human growth-hormone (hGH)	Varied	Hypertension, elevated malignancy risk	Hypertension
Diuretics	Furosemide, hydrochlorothiazide, torsemide	Reduce water retention to improve perceived muscle aesthetics usually taken before competition	Electrolyte disturbances, especially hypokalaemia
SARMs	Andarine, Ostarine, Ligandrol	Increase lean muscle mass	Unknown (experimental compounds)
Human chorionic gonadotropin (hCG) [47]	Varied	Counteract AAS suppression of testicular function and volume, raise testicular testosterone production	Diabetes, cardiomyopathy, renal failure, hepatotoxicity, edema, carpal tunnel syndrome, joint pain, fatigue
Site enhancement oil	Water-based, oil-based, and silicone-based injection options	Improve perceived aesthetics of muscle by locally expanding volume	Infection
Creatine [48]	Varied	Increase performance in short-duration, high-intensity exercises	Water retention, gastrointestinal symptoms, fatigue diarrhoea, liver and renal complications

**Table 4:** Commonly encountered side effects in the “postcycle” period.

Side effect	Examples of self-initiated therapies	Possible mechanisms	Important findings
Gynecomastia [49]	SERMS (tamoxifen) AIs (anastrozole, letrozole, exemestane) Dopamine agonists (cabergoline and bromocriptine) for galactorrhea	SERMs inhibit pituitary E2 receptors, and therefore stimulate pituitary gonadotropin release and subsequent endogenous testosterone secretion Aromatase inhibitors reduce the conversion of testosterone to estrogens, which exert powerful negative feedback on the HPT axis	Tamoxifen may effectively treat acute gynecomastia [51] Chronic gynecomastia may only respond to surgical treatment AAS users are also known to prophylactically administer SERMS and AIs to avoid developing gynecomastia
ASIH causing testicular atrophy, infertility, and low endogenous testosterone levels [46][	hCG injections (on-cycle) hCG injections (postcycle) SERMs (clomiphene, raloxifene) AIs (anastrozole, letrozole, exemestane)	The human placenta normally produces hCG, although synthetic forms are also available for exogenous administration hCG and LH bind to the same LH receptor [52] Serum and intratesticular testosterone levels can rise following hCG injections [53]	There is limited case report data demonstrating efficacy in accelerating return to endogenous testosterone production and spermatogenesis [54, 55]
Sexual dysfunction (low libido, erectile dysfunction) [49]	PDE-5 inhibitors (sildenafil, tadalafil) SSRI (dapoxetine) Herbal remedies Dopamine agonists (cabergoline)		Commentators have suggested PDE-5 inhibitors as the first-line treatment and discouraged herbal remedies and dapoxetine use [45]
